# Opportunity costs and local health service spending decisions: a qualitative study from Wales

**DOI:** 10.1186/s12913-016-1354-1

**Published:** 2016-03-25

**Authors:** Sarah Karlsberg Schaffer, Jon Sussex, Dyfrig Hughes, Nancy Devlin

**Affiliations:** Office of Health Economics, 7th Floor, Southside, 105 Victoria Street, London, SW1E 6QT UK; RAND Europe, Westbrook Centre, Milton Road, Cambridge, CB4 1YG UK; Centre for Health Economics and Medicines Evaluation, Bangor University, Ardudwy, Normal Site, Holyhead Road, Bangor, North Wales LL57 2PZ UK

**Keywords:** Opportunity cost, Priority setting, Cost-effectiveness, Health technology assessment, National Health Service, All Wales Medicines Strategy Group, National Institute for Health and Care Excellence

## Abstract

**Background:**

All health care systems face the need to find the resources to meet new demands such as a new, cost-increasing health technology. In England and Wales, when a health technology is recommended by the National Institute for Health and Care Excellence (NICE), the National Health Service (NHS) is mandated to provide the funding to accommodate it within three months of publication of the recommendation. Identifying what, in practice, is foregone when new cost-increasing technologies are introduced is important for understanding the effects of health technology assessment (HTA) decisions on the NHS or any other health care system. Our objective was to investigate how *in practice* local NHS commissioners in Wales accommodated financial “shocks” arising from technology appraisals (TAs) issued by NICE and from other cost pressures.

**Methods:**

Semi-structured interviews were conducted with Finance Directors and Medical Directors from all seven Local Health Boards (LHBs) in NHS Wales. These interviews covered prioritisation processes, as well as methods of financing NICE TAs and other financial shocks at each LHB. We then undertook a systematic identification of themes and topics from the information recorded. The study relates to the period October 2010 to March 2013.

**Results:**

The financial impact of NICE TAs is generally anticipated and planned for in advance and the majority of LHBs have contingency funds available to cope with these and other financial shocks within-period. Efficiency savings (defined as reductions in costs with no assumed reductions in quality) were a source of funds for cost pressures of all kinds. Service displacements were not linkable to particular NICE TAs and there appears to be a general lack of explicit prioritisation activities. The Welsh Government has, on occasion, explicitly or implicitly acted as the funder of last resort.

**Conclusions:**

Services may be displaced as part of a response to the cumulative impact of all types of cost pressures, including cost-increasing health technologies recommended by NICE, but such displacements were not direct responses to the publication of individual NICE TAs. The additional cost pressure represented by a new NICE TA is likely to be accommodated at least partly by greater efficiency and increased expenditure rather than displacement of services.

**Electronic supplementary material:**

The online version of this article (doi:10.1186/s12913-016-1354-1) contains supplementary material, which is available to authorized users.

## Background

Set up in 1999, the National Institute for Health and Care Excellence (NICE) provides national guidance and advice to improve health and social care. A key part of NICE’s role is conducting technology appraisals (TAs), where it makes recommendations on the use of new and existing treatments by the National Health Service (NHS) in England and Wales. The decision to recommend a technology is determined by both clinical and economic evidence, where cost-effectiveness is generally measured as the additional cost per quality-adjusted life year (QALY) gained, with some adjustments made to account for social value judgements such as the severity of underlying illness, disadvantaged populations or end-of-life treatment [1–3]. NHS commissioners of health care for their local populations are mandated to provide the necessary funding to meet such new recommendations by three months from the publication of the TA.

In practice, NICE bases its recommendations on a comparison of the incremental cost-effectiveness ratios (ICERs) of new technologies against a cost-effectiveness “threshold”. NICE has stated that its cost-effectiveness threshold lies in the range of £20,000–30,000 per QALY gained. As the ICER of a new technology increases above £20,000, explicit consideration of factors other than cost-effectiveness is required, and above £30,000 an increasingly strong case is needed with respect to these other considerations [2]. Dakin et al. [4] have shown that NICE’s past recommendations, although driven largely by the ICER, include many with ICERs well above £30,000 per QALY, reflecting other criteria considered by NICE.

The threshold range stated by NICE has been the subject of controversy. The House of Commons Select Committee on Health concluded in its 2007–2008 inquiry that “the affordability of NICE guidance and the range, measured in cost-per-QALY, it uses to decide whether a treatment is cost-effective is of serious concern. The threshold it employs is not based on empirical research and is not directly related to the NHS budget, nor is it at the same level as that used by PCTs [Primary Care Trusts – the territorial organisations responsible for purchasing health care for their local populations in England] in providing treatments not assessed by NICE” [5].

There have been various attempts to estimate the value of the cost per QALY threshold. One model, first put forward by Culyer et al. [6], involves identifying the threshold that lies between the least cost-effective technology currently provided and the most cost-effective technology not yet available routinely in the NHS. Appleby et al. [7] and Karlsberg Schaffer et al. [8] follow this model and use a bottom-up approach to attempt to estimate the threshold by identifying these marginal services and their corresponding costs per QALY gained. Claxton et al. [9] instead take a top-down approach to estimating the threshold: they use aggregate data on spending and outcomes by NHS PCTs across 23 Programme Budget Categories to estimate the average relationship between money spent and QALYs gained when comparing across PCTs.

Underpinning these attempts to estimate the cost per QALY threshold, and the health technology assessment (HTA) process itself, is an important assumption: that the approval of new, cost-increasing services will displace funds from existing health care services. If this assumption holds, the opportunity cost of NICE’s recommendation then depends on the ICER of the displaced service. The assumption is explicit in NICE’s decision making: “a technology can be considered to be cost effective if its health benefits are greater than the opportunity costs of programmes displaced to fund the new technology, in the context of a fixed NHS budget” [2].

To our knowledge, there has been no research that investigates the validity of the “displacement assumption” in the NHS. If it does not fully hold in practice, this has important implications for health policy and future research concerning the cost-effectiveness threshold and the opportunity cost of spending decisions. In particular, this paper discusses two other responses to cost-increasing HTA recommendations:

1. If health care providers are not yet getting the most from their resources, i.e. there is some so-called “x-inefficiency” [10] in the way they produce health care, then increased pressure of demand may be met to some extent by increased technical efficiency rather than by displacement of other health services.
2. If local health care organisations treat their budgets as, *in extremis*, not absolutely fixed and/or have the opportunity to turn to a funder of last resort, then some increased demand pressure may lead to increased expenditure, in which case the opportunity cost might fall outside the health budget. It is impossible for a local health organisation to have spent all of its annual budget to the last pound, and not a pound more, at midnight on the last day of the financial year. Thus every such organisation will have a non-zero underspend or overspend every year. Its response to increased pressure on its budget may in part be to underspend less or overspend more. It may even explicitly request increased funds from the Government. In Wales the funder of last resort for local health organisations is the Welsh Government, which may find the funds either from another part of the NHS in Wales or from other services for which it has responsibility (social services, education, etc.).

This paper fits within a broad area of recent literature from the UK and elsewhere that focuses on priority-setting and rationing of health care services. For example, Robinson et al. [11] is a qualitative research study investigating local priority-setting and resource allocation activity across five English PCTs. The authors note the “political complexity” involved in implementing the redesign of services and the lack of resources available to produce and understand cost-effectiveness evidence. Other issues highlighted in the priority-setting literature include the need for procedural justice and “fair” decision-making processes, shortages of local quantitative data to inform decision-making and the importance of leadership in the context of making “tough decisions”.

The aim of the current paper is to identify how NHS organisations, which might be considered to have fixed budgets, reallocated resources in practice when responding to a legal requirement to fund new, cost increasing technologies. Specifically, our objectives are to investigate: (a) how local NHS commissioners accommodated financial shocks arising from NICE TAs and from other requirements; and (b) how prioritisation decisions were made in the NHS by those budget holders.

## Methods

### Scope

Since October 2009, NHS Wales has been organised in seven Local Health Boards (LHBs) that are each responsible for commissioning and delivering all NHS health care services within a geographical area. The degree of organisational stability since 2009 made NHS Wales a more practical subject of research than any part of England, which was once again subject to a major reorganisation in 2012–2013.

Our study takes as its focus how the LHBs in Wales responded to the publication of cost-increasing NICE recommendations. NICE’s TA recommendations constitute mandates to the LHBs as they are required to have the necessary funds available for recommended services by three months from the date the recommendation is published by NICE. We asked senior medical and finance managers how their LHBs had accommodated NICE mandates arising between October 2010 and March 2013, allowing one year for adjustments to the NHS Wales reorganisation that produced the current LHB structure. The time period to which these data relate coincides with an era of reductions in health budgets for NHS Wales: over the period from 2010/11 to 2012/13 NHS spending per head of population fell by 1 % p.a. in cash terms in Wales [12].

### Official documents

At the start of the project we undertook Google searches, and searches of each LHB’s website specifically, to obtain any public LHB documents mentioning how any NICE mandate had been responded to. We found no such documents, and none was subsequently identified by any of the interviewees.

### Interview targets

We undertook a qualitative analysis based on semi-structured telephone interviews. The same questions, in the same order, were asked of all interviewees and the questions were open-ended rather than closed. We sought to avoid imposing assumptions on how or what the respondents would answer and were careful to ask neutral, non-leading questions. A copy of the interview questions is attached in Additional file 1.

To recruit interviewees for the study, we attended and presented our research objectives to All Wales meetings of Medical Directors in June 2013 and of Finance Directors in July 2013. We then approached the Medical and Finance Directors from each of the seven LHBs individually by email and asked whether they would be willing to participate in the study. Of the 14 Directors approached, 10 agreed to be interviewed or nominated a senior member of staff to be interviewed in their place. Three declined to be interviewed and one did not respond after two reminders. We interviewed at least one representative from every LHB (see Table 1): five Medical Directors and five Finance Directors. Interviews last between 21 and 46 min.

**Table 1:** Study interviewees

LHB	Medical Director	Finance Director
1	✓	✓
2	✓	✓
3	✓	✓
4	✘	✓
5	✓	✘
6	✘	✓
7	✓	✘

✓Individual was interviewed

✘Individual was not interviewed

### Interview structure

The purpose of the research was summarised at the outset of each interview, and the interviewer explained that no remarks would subsequently be attributed either to individuals or to particular organisations. Permission to audio record the interview was sought, and given in every case. The purpose of the audio recording was to enable the interviewer to confirm the accuracy of their notes of the points made in the interview. To further ensure accuracy, these notes were then sent to the interviewee for them to amend if necessary. The results presented in this paper are from the resulting agreed, validated notes of the 10 interviews.

The interviews were divided into three sections, as follows:

1. Planning and prioritisation
2. NICE Technology Appraisals
3. Other financial “shocks”.

In the first section of the interview, participants were asked about procedures, policies and guidelines for prioritisation at their LHB. This included information on the general process by which the costs of NICE TA recommendations are absorbed and whether LHBs had funds set aside especially for the implementation of NICE guidance.

In the second section, we asked the interviewees how *in practice* their LHBs had found the funds to comply with the particular NICE TAs that had been issued since October 2010. We requested information on any TAs that had a particularly large financial impact and how this impact was accommodated within a fixed budget. We also asked interviewees to identify any services which might have been displaced, in the sense that they were discontinued, received less funding, or the referral thresholds were significantly raised, in response to the financial burden imposed by a NICE TA.

In the third section of the interview, we focussed on financial shocks other than the required implementation of NICE recommendations. We asked participants to explain how, in general, they accommodated shocks and to identify any that were particularly problematic. We also asked how savings were made within each Board and whether this entailed displacement of services, delays in planned increases, making efficiency savings or allocating contingency funds.

### Analysis

Nine of the interviews were conducted by the same researcher (SKS). Consistency in information recording between those and the tenth interview (by JS) was ensured by the common questionnaire scripts being used (Additional file 1), by the two researchers reading and commenting on each other’s interview notes, and by the process of confirming the notes with the interviewees themselves.

When the 10 interviews were complete and the corresponding sets of notes were available, SKS and JS independently reviewed them and independently undertook systematic (coded) identification of themes and topics from the information recorded. The two researchers then compared their respective analyses. Where the researchers were initially unsure of their interpretations of an interviewee’s response on a particular point a consensus was reached by returning to the source material together.

All interviewees were asked to highlight any key documents relevant to our questions. Where documents were identified we either obtained these directly from LHB or other organisations’ websites or they were provided by the interviewee.

A Consolidated Criteria for Reporting Qualitative Studies (COREQ) 32-item checklist is available in Additional file 2. 

## Results

The results of the interviews are summarised in Table 2, where themes and topics identified by the interviewers/analysts are listed in the first column. LHBs are labelled 1–7 and in a random order to protect anonymity. At the three LHBs where we obtained two interviews there were no inconsistencies between the responses we received from the separate respondents.

**Table 2:** Summary of results

	Local Health Board						
	1	2	3	4	5	6	7
Institutional frameworks for prioritisation							
Framework for prioritising interventions	✘	✓	✘	✘	✓	✘	✘
Interventions Not Normally Undertaken	✓	✓^a^	✓	**∙**	✓	✓^a^	✓^a^
Responding to NICE TAs							
Horizon scanning	✓	✓	✓	✓	✓	✓	✘
NICE contingency fund	✓	✓	✘	✘	✓	✓	✓
Efficiency savings	✓	✓	✓	✓	✓	✓	**∙**
Displacements linked to individual NICE TAs	✘	✘	✘	✘	✘	✘	✘
Other examples of displacement by LHB	✘	✓	✘	✘	✓	✘	✘
Phasing in of NICE guidance	✓	**∙**	**∙**	**∙**	**∙**	✓	✓
Savings first sought in same clinical programme	✘	✘	✘	✘	✓	✘	✓
Savings first sought in medicines budget	✘	✓	✘	✓	✘	✘	✘
Extra funds sought/received from Welsh Govt.	**∙**	**∙**	**∙**	✓	**∙**	**∙**	✘
Responding to other financial shocks							
Contingency fund for other shocks	✘	✓	✘	✘	✓	✓	**∙**
Extra funds requested/received from govt.	✓	**∙**	✓	**∙**	✓	**∙**	✘

^a^Not referred to in interviews but found online by authors

✓Topic was mentioned by either one or two interviewees from that LHB

**∙** Topic was not referred to specifically

✘Interviewee confirmed that the practice did not occur in their LHB

LHBs are labelled 1–7 and in a random order to protect anonymity

The remainder of the Results section discusses these topics in turn, grouping them under the following two main headings:

- Institutional frameworks for prioritisation
- Responding to NICE TAs and other financial shocks.

### Institutional frameworks for prioritisation

#### Frameworks for prioritising interventions

The Medical Director from LHB 5 explained that the development of prioritisation frameworks had been discussed at an All Wales level and that each Board was expected to create its own Prioritisation Panel. However, their development appeared to be at only the beginning stages across most of Wales.

Interviewees from five of the seven LHBs (1, 3, 4, 6 & 7) said that their LHB had no formal method for prioritising the services provided. The Finance Director from LHB 6 explained that although their LHB has a panel that reviews NICE guidance, they do not have a process that would be “recognised as mechanistic or formulaic” for testing the value for money of interventions and the panel does not manage services other than those recently appraised by NICE. The Finance Director from LHB 1 commented that “the level of clarity … is not yet such that decision-makers assess the marginal benefit of various procedures versus those which NICE is recommending”.

The Medical Director from LHB 1 explained that in the event of numerous groups of clinicians requesting that various additional treatments are funded, each group would produce a business case which would be required to map back to priorities outlined by the Welsh Government.

One LHB has created a Prioritisation Panel to examine potential service developments and disinvestment opportunities (LHB 5). At this LHB, the criteria used by the panel to prioritise service developments include: clinical effectiveness, cost-effectiveness, and equity and equality impact. The Medical Director said that cost-effectiveness can be difficult to assess, given that formal health economic evidence is often missing. Examples of decisions the panel has made so far include determining the referral thresholds for cataract surgery and using lifestyle interventions to maximise the potential from hip and knee operations.

At this same Board, an exercise was undertaken to identify potential areas for disinvestment by Clinical Programme Group (CPG). The resulting document lists procedures currently on the NICE “do not do” list as well as the Cochrane Quality and Productivity Topics, where the latter “highlight potential disinvestment opportunities that can be used by the NHS to meet its … targets, and include calculations of potential cost savings if implemented.”

At LHB 2, a Prioritisation Board and a Clinical Effectiveness Board have been developed. At the time of interview, the Clinical Effectiveness Board had heard a number of cases but the Prioritisation Board had, we were told, been little referred to. An explanation given by the Finance Director for the limited use of the Prioritisation Board is that the LHB “very rarely does *proactive* investment” – most of their investments are obligatory, e.g. staff pay-awards. They also have an Equalities Impact Assessment (EQIA) system in place which is used to predict the impact of planned disinvestments such as reductions in staff and bed numbers. For example, the LHB carried out an EQIA of plans to close their Inpatient Mother and Baby Unit – the net impact was found to be positive due to the reinvestment of the funds. In assessing potential disinvestments, cost-effectiveness evidence is used but this not in terms of QALYs.

Another interviewee (Finance Director) said that their LHB (4) was likely to develop a formal prioritisation framework in the coming years.

Thus, overall, no respondent identified their LHB as yet using an explicitly documented framework of criteria for prioritising expenditure decisions, such as when required to find funds for a new NICE mandate.

#### “Interventions not normally undertaken”

We were alerted by two interviewees to an All Wales policy document called “Making Decisions on Individual Patient Funding Requests (IPFR)” [13]. Its purpose was to improve transparency around the availability of treatments provided by the NHS. In particular, the document outlined the policy in NHS Wales for requests which fall outside the range of services routinely provided by LHBs and the Welsh Health Specialised Services Committee (WHSSC).

As is explained on p.3 of the document, such requests will normally be within one of the three following categories:

- A treatment that is either new, novel, developing or unproven and is not within the LHB’s routine schedule of services and treatments
- A treatment that is provided by the LHB in certain clinical circumstances but is not eligible in accordance with the clinical policy criteria for that treatment
- A rare or specialist condition that falls within the service remit of the WHSSC but is not eligible in accordance with the clinical policy criteria for treatment.

The document provides a guide for LHBs to dealing with IPFRs, where the core principle is that the requests must demonstrate “exceptionality”. There was no mention of an equivalent guide to non-exceptional expenditure decision-making.

As detailed in the All Wales policy on IPFR, all LHBs were required to compile a list of Interventions Not Normally Undertaken (INNU). Three LHBs (1, 3 & 5) referred to it during the interviews and INNU lists for three other LHBs were found in subsequent Google searching. The reasons for an LHB not normally providing a service may be one of the following three (as described in the All Wales IPFR document):

- There is currently insufficient evidence of clinical and/or cost effectiveness
- The intervention has not been reviewed by NICE or the All Wales Medicines Strategy Group
- The intervention is considered to be of relatively low priority for NHS resources.

Having examined all available INNU policies (six), it is clear that the level of detail and the use of evidence vary from Board to Board. In the INNU document for one LHB, economic considerations are addressed specifically:

“The [LHB] can no longer consider investing in any new developments unless they are clearly more effective, improve patient experience and health outcomes, and are at least equal in value for money to existing services or interventions. Choosing one intervention or service means that the [LHB] cannot provide another – that is, there are opportunity costs to everything that [the LHB] does.”

Many of the interventions listed are common across boards. Some examples of interventions not normally undertaken across much of NHS Wales include:

- Cosmetic procedures
- Orthodontic treatment
- Sterilisation reversal
- Tattoo removal
- Varicose vein treatment.

### Responding to NICE TAs and other financial shocks

#### Horizon scanning and contingency funds

The way in which LHBs manage the implementation of NICE TAs varies from Board to Board. All but one LHB (LHB 7) mentioned “horizon scanning”: a process that determines which medicines or other technologies are likely to be recommended in the coming financial year and the potential impact this will have on their finances. In five of the seven LHBs (LHBs 1, 2, 5, 6, & 7) this is linked to the creation of a NICE contingency fund, set aside specifically to accommodate the estimated financial burden expected from new NICE mandates during the coming year. Some of the LHBs also mentioned contingency funds for other specific areas of financial uncertainty (as distinct from a single common contingency for all financial shocks).

The horizon scanning process is often done “in house” by LHBs’ pharmacy/medicines departments but it builds on national level horizon scanning by NHS Wales pharmacists who provide each LHB with their estimate of the local impact, given the LHB’s population size and characteristics. Depending on the LHB, either it decides to set exactly that amount aside (2/7 LHBs), or it instead uses the centrally provided estimate as merely a guide and it may perform its own horizon scanning exercise (5/7 LHBs).

When asked specifically how their LHB dealt with NICE TAs that were unexpected and so could not have been planned for during the horizon scanning process, we received varied responses. Interviewees from three LHBs (1, 2 & 5) were adamant that their LHB planning process had ensured that their Boards had not been caught out by NICE mandates in the period since October 2010. Both interviewees from LHB 1 stated that their LHB’s contingency fund had always proved to be larger than was necessary. The Finance Director from LHB 2 said that NICE timescales tend to “slip” leading the LHBs to overestimate the in-year impacts of NICE TAs.

Despite the level of planning that takes place, we were told of instances where NICE TAs had been problematic for LHBs to accommodate. The following TAs were mentioned by interviewees when asked for any examples that had had a notable local financial burden:

- Rivaroxaban (2012) – a new anticoagulant (3)
- Boceprevir and telaprevir (2012) – new hepatitis C protease inhibitors (3)
- Aflibercept (2013) – drug for age-related macular degeneration (AMD) (3)
- Golimumab (2012) – monoclonal antibody therapy for rheumatoid arthritis (2)
- Omalizumab (2013) – a recently reviewed asthma drug (1).

The year of recommendation and the number of LHBs mentioning the TA are in brackets.

#### Efficiency savings

The most common response to the question of how cost-increasing NICE TAs are accommodated involved the LHB making efficiency savings, that is reductions in costs which are intended not to lead to reductions in benefits. The demands of NICE TAs are in all LHBs considered as one of a number of “cost pressures” that the Board must deal with in-year. Other cost pressures include increased demand for services, staff pay awards and energy price increases.

From LHB 2, we were able to identify some other examples of financial shocks. These included problems with cardiac surgery that forced them to outsource a number of patients to England and the previous year’s “bad winter”, which caused them to cancel operations and therefore incur extra costs to meet waiting time targets. The interviewee also mentioned that their LHB had been expecting to benefit from savings from the costs of generic (“Category M”) medicines but these were not realised because the procurement arrangements were changed. In total, this LHB faced unexpected cost pressures in-year equivalent to approximately 1 % of its total annual budget.

We were told by all that it was the *totality* of these cost pressures which force the LHBs to seek efficiencies or other savings. The Finance Director from LHB 1 explained that potential savings are broken down into different categories such as: workforce productivity, service redesign, non-pay operating expenditure (general supplies and services), management and procurement.

More specifically, the Finance Director from LHB 6 named reducing staffing costs and bed numbers and treating patients as day cases rather than inpatients (where appropriate) as specific means of making savings. With regards to reducing staffing costs, the Finance Director from LHB 4 said that the focus was on control of *variable* pay (pay for agency staff and overtime), as opposed to that of core staff. The same interviewee also described efficiency savings in the LHB’s use of medicines, including switching from branded to generic drugs and not issuing prescriptions for medicines that can be bought over the counter.

It should be noted that a distinction can be drawn between pure efficiency savings (reductions in x-inefficiency or “slack”), such as those resulting from substitution to generic drugs, which can reasonably be assumed to have no impact on health outcomes, and efficiency savings where the impact on health is more complex. For example, when the Finance Director from LHB 4 was asked whether staffing cuts were expected to affect the quality of service provision, they explained that staff safety levels on wards were always maintained but there could be longer waiting lists (for elective surgery, for instance) as an unintended consequence of achieving savings. This is an example of where what is described as an efficiency may nonetheless have an opportunity cost in terms of forgone benefits to patients.

The Finance Director from LHB 2 explained that areas in which efficiency savings can be made are identified using a benchmarking process. This involves comparing statistics across a range of metrics (average length of stay, day case rates, waiting times, etc.) between their own LHB and other Welsh LHBs, as well as NHS providers across England.

Another participant, the Finance Director from LHB 1, provided an interesting insight into the importance of efficiency savings as opposed to service displacements when responding to cost pressures of any kind. They explained that the tendency to make efficiency savings rather than to reduce the provision of services on the basis of cost-effectiveness is the result of the fact that the commissioning function in NHS Wales has “waned with the internal market” (which was abolished in 2009). In other words, because the purchaser-provider split that previously existed in Wales no longer exists, decision makers are now less focussed on which services to continue or discontinue. The interviewee added that “looking at *what* we provide as opposed to the *efficiency* of what we provide is less focussed [in Wales]”.

We were told on numerous occasions during the interviews that making efficiency savings was particularly important given that NHS Wales has been operating in a “flat cash” environment in recent years, resulting in a real terms reduction in funding after accounting for input price inflation. This is a challenging environment in which to accommodate cost pressures: one interviewee described how their LHB had to be “continuously improving efficiency throughout the organisation” to cope (LHB 2, Medical Director). Another explained that all Welsh LHBs were forecasting a deficit against budget for the current financial year (2013/14) (LHB 1, Finance Director).

#### Displacement of health services

We asked interviewees directly whether their LHBs had since October 2010 been forced to disinvest from health improving services in order to meet the funding demands imposed by a newly published NICE TA. In all cases, the interviewees could recall no examples where this type of direct displacement had taken place. The Medical Director from LHB 7 explained that he could not think of a single occasion where an “either, or” decision had to be made. In addition, two Medical Directors highlighted the absence of guidance on how displacement could be achieved (LHBs 5 & 7).

A common theme throughout the interviews was that a Board may have to make planned reductions in services, though not necessarily to health-improving services, but that it would not be possible to link such displacement to any one cost pressure, let alone any specific appraisal. A specific example given of a planned cost saving was the cancelling of an outreach clinic at a local venue to save costs, meaning that patients would have to travel further to obtain the service at a hospital (LHB 5, Medical Director).

One interviewee explained that although their LHB had not made disinvestments in response to the financial burden of specific NICE TAs, the *cumulative* cost of TAs through the financial year had led them to delay desired investments (LHB 2, Finance Director). The principle of delaying a desired investment is similar to that of making an explicit disinvestment in that both can be expected to have a detrimental effect on population health relative to their respective counterfactuals. It is important to note, however, that delaying investment in new or expanded services may be regarded as more acceptable in terms of public/political perception relative to cutting existing services.

The Finance Director from LHB 2 gave the example of fidaxomicin, an antibiotic for the management of *Clostridium difficile* that they would like to prescribe because it has the potential to reduce instances of health care acquired infections, but which they do not offer because they do not have the funds. The interviewee stated that there was a “strong link” between the cumulative pressure of NICE TAs and the decision not to invest in the antibiotic but that it was not the only factor, and this displacement could not be linked to any individual TA. Other examples of delayed investments that can be linked to the total financial burden imposed by NICE TAs include the replacement of medical equipment and the modernisation of the LHB’s IT/prescribing system.

At LHB 7, the Medical Director explained that, although they could not think of a specific example, implementing NICE TAs would be likely to displace some “normal activity”, perhaps resulting in increased waiting times for some patients.

#### Phasing in of NICE guidance

Respondents at three of the seven LHBs offered an additional type of response to the question of how LHBs accommodate unexpected NICE TAs, namely by delaying their response, at least in part, beyond the date three months from the publication of the NICE TA (LHBs 1, 2 & 7). Delaying the response to a new NICE mandate correspondingly delays the point at which responding to the mandate generates opportunity costs. It was pointed out that some NICE mandates require prior build-up of infrastructure – appropriately trained staff, perhaps additional diagnostic services, which are not initially available in sufficient quantities – meaning that the rate of implementation of the mandate is inevitably less than immediate and is to a degree at the discretion of the LHB concerning the speed of build-up.

For example, the approval of boceprevir and telaprevir (hepatitis C protease inhibitors) was very costly to implement in parts of Wales due to the high number of patients eligible for treatment. The Medical Director from LHB 7 explained that the infrastructure simply did not exist to administer the drugs to every eligible patient in their area of responsibility from day one. Instead, they started treating the neediest patients first and created a plan so that all eligible patients would be treated within 6–9 months. Another interviewee (LHB 1, Finance Director) explained that because all patients must be assessed before they can receive the hepatitis C drugs, their LHB began running the assessment clinic within the three month window but did not start treating patients until later.

In a similar manner, the Finance Director from LHB 6 said that had they implemented the guidance for rivaroxaban to its fullest extent as soon as it was published, this would have had a “crippling” effect on the LHB’s finances. Instead it was introduced in a “more constrained” manner.

#### Where the opportunity cost arises

**Fig. 1:**
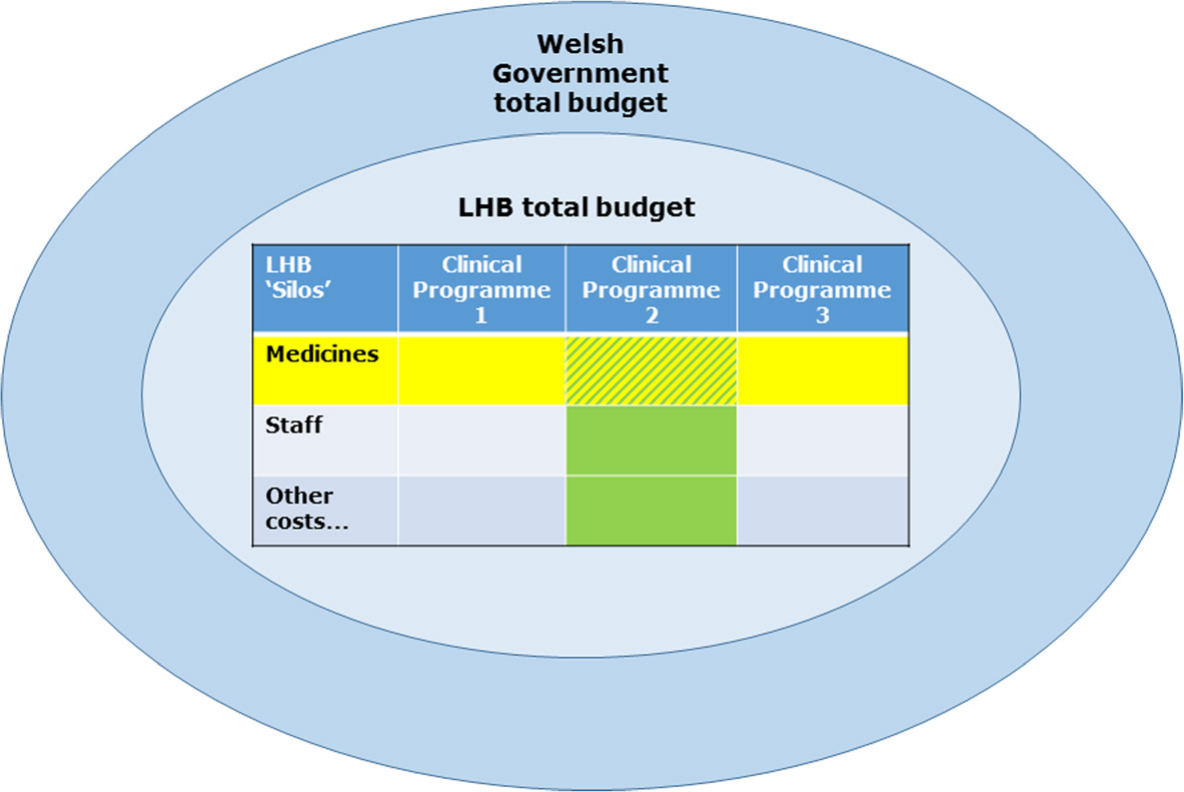
Where opportunity costs might arise. Figure 1 illustrates schematically the range of possibilities for where the opportunity cost of new TAs may lie. Offsetting savings may be found in a particular clinical programme (shown as columns) or in another type of expenditure such as the LHB’s medicines bill or staff costs (shown as rows). The yellow/green shading in Fig. 1 illustrates the example of an LHB finding offsetting savings in the medicines budget of Clinical Programme 2

Our interviews revealed different approaches in different LHBs to where funds would be sought in response to the need to deliver a new NICE mandate. Figure 1 illustrates schematically the range of possibilities. An initial response might be to look for an offsetting saving in the same clinical programme (cancer, mental health, etc.) to which the new NICE TA applies (shown as a shaded column of the matrix in the centre of Fig. 1). Elsewhere the first target might be the LHB’s medicines bill – as opposed to any other type of expenditure such as staff costs (a shaded row of the matrix in the centre of Fig. 1). Our interviews revealed two LHBs that look first for offsetting savings within the same clinical programme area (LHBs 5 & 7), and two that look first within the LHB’s medicines budget (LHBs 2 & 4).

An LHB might not take such a “silo” view of its expenditures and instead look across the whole of its budget for possible offsets to the new cost pressure. Three of the LHBs in Wales appear from our interviews to take this approach at the outset (LHBs 1, 3 & 6).

If the LHB failed to find the funds itself it might either increase its spending by reducing a planned underspend or taking the risk of a greater overspend of its budget – bearing in mind that the cost of an individual NICE TA is typically considerably less than 1 % of an LHB’s total annual budget – or it might actively seek additional funding from the Welsh Government. In that case the opportunity cost might either fall on some part of the NHS outside the LHB receiving the additional funds, or on a non-health care part of the Welsh Government’s expenditure.

Some interviewees highlighted occasions where their LHB had been hit by a financial “shock” (from a NICE TA or elsewhere) and had been able to request extra funds from the Welsh Government to accommodate it. For example, the Medical Director from LHB 5 explained that when the guidance was issued for an Age-Related Macular Degeneration (AMD) drug, it amounted to a “substantial additionality that was very difficult to absorb”. As the Welsh Government considered the drug an “irrefutably beneficial technology”, they helped the LHB by paying a contribution towards infrastructure costs and the unit costs of the drug. It was argued that doing so would create long-term savings in social care. Disinvesting in other areas of ophthalmology such as cataract surgery was not seen as an option because there was high demand for these procedures as well.

Occasions other than new NICE TAs where LHBs have requested extra funds from the Welsh Government to cover unexpected costs include an emergency refurbishment of a hospital due to the use of asbestos (LHB 5) and meeting increased vaccination costs in response to a recent measles epidemic (LHB 1). In general, situations where the Welsh Government might be expected to absorb cost pressures are those which pose a national public health risk.

In addition, one interviewee explained that their LHB has typically overspent on its total budget in recent years, and anticipated this at the start of each financial year (LHB 3, Finance Director). The amount overspent each year has been funded by the Welsh Government from other NHS Wales funds: either requiring repayment the following year or writing off the amount.

## Discussion

The cost per QALY threshold range applied by NICE, the Scottish Medicines Consortium (SMC) and the All Wales Medicines Strategy Group (AWMSG) is £20,000–£30,000 per QALY gained, implying that when a technology is appraised, it is more likely to be approved if its cost per QALY is at or below this level [2, 14, 15]. These recommendations are based on the assumption that the objective of the NHS is to maximise health gain, which in practice NICE measures in terms of QALYs, with some adjustment of the £/QALY threshold to account for social value judgements [3].

LHBs’ budgets are assumed to be fixed and fully deployed, in the sense that available budgets are fully allocated to health care and are exhausted at the end of each time period. In addition, health care providers are assumed not to be x-inefficient, so that newly approved technologies will displace services currently in operation rather than being able to be funded from efficiency improvements. Our research indicates that all of these assumptions are questionable.

### Maximising health gain

We found that none of the LHBs in Wales currently makes routine use of an explicit framework for prioritising expenditure decisions. *A fortiori* there seems to be no explicit decision making framework in use aimed at maximising health gain, measured by QALYs or in any other way, or any combination of that with other objectives.

Expenditure decisions in Wales are clearly strongly affected by the range of health services currently provided, and by a desire not to reduce any existing services if at all possible, despite real-term reductions in budgets.

### Planning

For the most part, it appears that, with the aid of horizon scanning at both a local and national level, LHBs in Wales successfully anticipate the financial scale of NICE TAs to be published over the coming financial year. Contingency funds are created and have, on most occasions, proved adequate in the period under study (2010–2013). In the HTA process, it is assumed that spending in one area (as follows from the recommendation of a new technology) necessarily involves shifting resources away from another area. However, if the other area is a contingency fund, this makes the identification of the opportunity cost dependent on a different stage of decision-making: the prioritisation of expenditures at the planning stage when the contingency fund is set. Nevertheless, if the creation of a contingency fund is achieved by not spending on health services, then its opportunity cost may be counted in terms of patient gains forgone.

The successful use of contingency funds has meant that our search for evidence of services displaced by medicines and other technologies newly mandated by NICE *as a short-term response to an in-year financial pressure* has yielded no examples of displacement that can be directly linked to individual NICE TAs. This implies that the strategy pursued by Karlsberg Schaffer et al. [8] in their recent study of the NHS Scotland is the more fruitful one in terms of identifying what services may be displaced at the margin. That study looked a Scottish Health Boards’ *plans*: what they planned to invest in at the margin, what they planned to delay implementing and what, in a small number of cases, they planned to disinvest from. The result was a list of services around the margin – but demonstrating a very wide range of costs per QALY.

### Efficiency

In most activities there is scope to improve efficiency and the language of NHS Directors focuses heavily on doing so. The interviewee from LHB 2 encapsulated this as “continuously making efficiency savings”. The assumption of economic models that providers produce services at any given volume and quality at minimum cost is a crude approximation (albeit one which has the merit of making the mathematics of such models much more tractable).

Our interviews revealed that when demand pressures increase – as a result of NICE mandating a new technology or for any other reason – but the budget available to meet them does not, an important part of the response is to try to squeeze out greater efficiency from providers. In the short term this may mean pay freezes or other cost-cutting, and in the medium term it may mean changing the ways in which services are provided. For example, the approval of an existing drug for a new patient group may result in increased demand for nurses’ time but instead of the LHB reallocating resources towards that clinical area, the existing nurses may simply be worked harder. The displaced “object” in this example could be considered to be “nurses’ spare time”, the opportunity cost of which is very difficult to quantify.

Efficiency improvements are a continuing and important source of funds to meet additional cost pressures of all kinds, including the cumulative impact of NICE TAs. This finding is consistent with the priority-setting literature from elsewhere in the UK: for example, there is evidence that in the NHS in England disinvestment is “distinctly counter-cultural” and that NHS providers are not familiar with “stopping doing things” [10].

If efficiency improvements would have been made to the same extent even in the absence of the financial shock, e.g. from a new cost-increasing NICE mandate, then using them to release funds to pay for that mandate implies that they are not being used to provide other health services, and so the opportunity cost may be thought of in terms of health gains forgone by others. However, to the extent that it is only the pressure for increased spending that stimulates the increased efficiency, which would not have occurred in the absence of that financial pressure, then there is no implied displacement of health services by the new NICE mandate and hence no foregone health gains elsewhere.

### Mutable budgets

Thus far, we have discussed the evidence that, in practice, a LHB’s objective function (i.e. a quantitative summary of its goals) may be undefined or contain more elements than QALYs alone and may not necessarily be maximised. An additional assumption implicit in the economic model underlying NICE’s TA process concerns the *constraint* in the optimisation problem: it is assumed that LHB budgets are “hard” in the sense that they are strictly enforced by central government; that they are fixed and unbreakable.

We have found that LHBs had some ability to increase their spending when faced with increased cost pressures, despite a legal duty to break even at the end of each financial year. This may be achieved by underspending less or overspending more against budget, given that outturn spend is unavoidably not identical to budget given the uncertainties in health care demand and costs. Or it may be achieved by explicitly requesting and obtaining additional funds from the Welsh Government. This is possible because the Government has the flexibility to increase its NHS spending at the expense of other programmes out of its block allocation from HM Treasury for devolved services. Thus the budget constraints facing LHBs (and indeed any other NHS and many non-NHS public bodies) may in fact be “soft” to some degree.

The possibility of a central government “hand-out” or “bail-out” has consequences for the incentives of NHS decision makers: the Welsh Government is acting as the “lender of last resort”. Kornai [16] provides a discussion of the efficiency losses that may result from the existence of soft budget constraints, focussing on the theory of the firm. In a specific application to health care, Shen and Eggleston [17] report evidence that the “softness” of budget constraints can affect quality improvement innovation and cost control in hospitals.

If NHS budgets in future years are assumed to be unaffected by NHS expenditure this year, then the respite for LHBs from increasing spending this year will be short-lived and they will have to plan to displace services or reduce x-inefficiency further next year rather than have access to increased funds from the Government. But if it is assumed that NHS spending in future years is determined in part by NHS expenditures in past years, then if LHBs increase their spending in response to a new NICE mandate or other increase in demand, the opportunity cost of that may lie outside the NHS and could be in any government sector. In that case, the opportunity cost is no longer in terms of forgone health gain.

### Policy implications

It is clear from our findings that the methods for deciding on investments in health care technologies in the NHS in Wales (NICE TAs) and those for accommodating those investments at the LHB level differ. The consequences of that difference for the overall health of patients in Wales are unknown. The apparent absence of a prioritisation framework for LHBs suggests an avenue worth considering would be to develop one. It may also be worthwhile to consider the implications for NICE’s approach of the criteria LHBs might wish to apply.

### Avenues for future research

An important question that emerges from our study is how to define the NHS’s objectives in a way that fits with the processes and decisions we observe in practice. One avenue to explore is the concept of “satisficing”: where individuals or organisations attempt to achieve at least some minimum level of a particular objective or group of objectives, but do not necessarily maximise any one, or any particular combination of them [18]. For example, the objective of an LHB could be to ensure that health (measured in QALYs or otherwise), waiting times and staff satisfaction each do not fall below (or above, in the case of waiting times) certain levels, but not the levels that maximise health. Further work could explore this, perhaps by combining quantitative analysis of time series data on a number of indicators of interest to LHBs with qualitative analysis such as interviews similar to those in the current study.

Another important direction that may be explored is the extent to which budget constraints are in practice treated as binding and the theoretical and practical implications of soft budget constraints. This behaviour is not restricted to Wales: across the UK health system we observe some health localities overspending their supposedly fixed budgets and others underspending by non-negligible amounts. Other parts of the UK public sector that are also subject to supposedly fixed budgets demonstrate similar flexibility.

### Limitations of the study

The material for our qualitative analysis was obtained via telephone interviews with senior managers of the seven LHBs in Wales. LHB Medical Directors and Finance Directors can be expected to have a clear but high level view of expenditure prioritisation and how such decisions are made. Inevitably they will be unaware of all the other decisions being made at lower levels within LHBs. We also cannot be certain that the information we received is representative of all decision makers in NHS Wales, including other Board members such as the Directors of Public Health and the Directors of Nursing, and All-Wales level decision makers such as those at Public Health Wales.

As with all interviews, there exists the possibility of recall bias by the respondents, either inadvertently or through caution. The guarantee of individual and organisation anonymity was intended to help encourage open and full responses to our questions, but our total sample was relatively small. The October 2010 to March 2013 time period we asked about was a compromise between being recent and hence reasonably fresh in respondents’ minds, but going far enough back to encapsulate a substantial number of cost-increasing NICE mandates.

Our method of questioning is not the only one that could have been used. For example, we might have presented interviewees with a hypothetical scenario, or a series of such scenarios, and asked them what their actions would have been, rather than asking them what actually happened in the past. However, our approach has the major advantage of grounding the responses directly in the respondents’ experiences: these were financial pressures that they and their colleagues had really faced and had actually dealt with. Thus the responses can be expected to reveal how much displacement took place and where, and hence where the opportunity cost would have arisen.

Our study was limited to Wales, for reasons of practicality (limited budget and timescale) and because Wales represented a part of the NHS that had, unlike England, not been the subject of disruptive organisational reforms in the last three years. We would expect that our findings should be of some relevance also to the NHS in England, Northern Ireland and Scotland. Funding levels differ across the four countries of the UK, as do the specific health care needs of their respective populations but at a broad level both funding and need may be reasonably assumed to be similar. The NHS in England has separate bodies for commissioning and providing health care, whereas in Wales and the rest of the UK both of these activities take place within the same overall organisations (LHBs in Wales). We cannot tell if this would lead local NHS commissioning bodies in England to respond to NICE mandates in systematically different ways from how LHBs in Wales do, but we have no reason to expect systematic differences.

## Conclusions

The principal aim of this research was to analyse what happened in practice when LHBs in Wales were required to fund cost-increasing health technologies as a result of NICE TAs. We searched for public domain documentation and interviewed 10 of the 14 Medical and Finance Directors of LHBs (or their nominees).

We found that the majority of NICE recommendations were anticipated and planned for by LHBs using horizon scanning. This process was often used to create contingency funds that had hitherto usually proved to be adequately sized to deal with the financial pressures that arose within a financial year. Therefore, the opportunity cost of accommodating NICE TAs is to a large extent determined at the LHB’s budget-setting stage, at the point where the size of the contingency fund is decided. This means that planned changes are important as a source of information about the opportunity cost of marginal expenditures in the NHS.

Services may have been displaced as part of a response (generally a planned response) to the cumulative impact of all types of cost pressures, but displacements were not linkable to individual NICE mandates that were published. To the extent that services were displaced to make funds available for new cost-increasing health technologies, then the opportunity cost may be estimated in terms of patient benefits, principally health gain, forgone.

When it was necessary to find additional funds to accommodate new NICE TAs and other cost pressures, this was generally achieved at least partly by improving efficiency. To the extent that these efficiency improvements would not have been sought in the absence of the increase in pressure on finances, and to the extent that the efficiencies were genuine reductions in x-inefficiency, meaning that the same quantity and quality of existing health care services is provided as before but at lower cost, then there are no forgone patient health gains. In order to find an offsetting saving, some LHBs looked within the same clinical programme as the NICE TA, while others looked within LHB “silos” such as the medicines budget.

A third type of response to a financial shock was simply to increase spending: by underspending the LHB budget by less, by overspending it more, or on occasion by explicitly requesting increased funds from the Government.

Overall we conclude that the opportunity cost of new, cost-increasing NICE mandates is not wholly felt in terms of displacement of other NHS services, but at least in part is reflected in increased efforts by health care providers that result in greater efficiency. On occasion there is increased NHS expenditure, which implies that part of the opportunity cost may fall outside the NHS. We hope that our findings can be used to strengthen the design of future research into the opportunity costs of introducing cost-increasing health care technologies.

## Additional files

Additional file 1: Interview structure. (DOCX 16 kb) Additional file 2: COREQ checklist. (DOCX 20 kb)

## Abbreviations

AMD: age-related macular degeneration
AWMSG: All Wales Medicines Strategy Group
CPG: clinical programme group
EQIA: equalities impact assessment
HTA: health technology assessment
ICER: incremental cost-effectiveness ratio
INNU: interventions not normally undertaken
IPFR: individual patient funding request
LHB: local health board
NHS: National Health Service
NICE: National Institute for Health and Care Excellence
QALY: quality-adjusted life year
SMC: Scottish Medicines Consortium
TA: technology appraisal
WHSSC: Welsh Health Specialised Services Committee
