# Supplementary material for: Opportunity costs and local health service spending decisions: a qualitative study from Wales
Source: BMC Health Serv Res. 2016 Mar 25;16:103. doi: 10.1186/s12913-016-1354-1 (PMC4807555; doi:10.1186/s12913-016-1354-1)
Supplement: Additional file 1: — Interview structure. (DOCX 16 kb) [file 12913_2016_1354_MOESM1_ESM.docx]

**Additional file 1: interview structure**

**Interview plan**

1. Give some definitions of important words/phrases
2. Provide a short recap of the project and the information we would like from you
3. Conduct a structured interview about spending decisions since October 2010
4. Opportunity for interviewee to ask questions

**Definitions**

- Technology = intervention recommended by NICE’s technology appraisals which the NHS is legally obliged to fund. These are mainly medicines, but also some medical devices
- QALY = quality-adjusted life year
- Marginal = added or subtracted, e.g. marginal spending = spending on new services or increased spending on existing services

**Background to the research**

- This is a joint project between the Office of Health Economics and Bangor University, funded by an unrestricted grant from the American Pharma Group (APG) and the Association of the British Pharmaceutical Industry (ABPI). We intend to publish a paper in a peer reviewed academic journal.
- NICE makes decisions about new technologies that NHS Health Boards in Wales and their counterparts in England are required to fund. The majority of NICE’s decisions entail cost increases as well as benefit increases, in aggregate across Wales amounting to a budget impact of millions of pounds in a year.
- NICE’s decisions are based on the assumption that the recommended technologies are better value for money than some other technologies that are funded in the health care system; and that when the recommended technologies are introduced, NHS Health Boards will be able to fund them by diverting resources from other services that are poorer value for money.
- *But no-one has ever done any research to check if that is actually the case.*
- We think this is a really important issue. For example, if the existing services that get displaced as a result of new technologies are, in reality, better value for money than the ones newly mandated by NICE, then patient health (overall) will be made worse, not better.
- We are focusing on actual decisions made in the period October 2010 to March 2013, i.e. since the implementation of the reorganisation that produced the current structure of the NHS in Wales.
- We do not approach this research with any agenda, or assumptions in mind about the conclusions. We just want to find out what goes on in practice and how decision makers like you cope when you have to suddenly find the resources to fund NICE decisions.
- Information revealed in this interview will be aggregated with that collected from the other interviews. In any publication no remarks will be attributed to any individual or organisation unless the individual concerned has given permission to do so.

**Interview**

Throughout the interview please inform us of any documentation we might read that records and explains the decision-making process and the decisions made.

Planning

- Are you aware of any procedures, policies or guidelines for prioritisation?
- Could you describe the general process by which the costs of NICE technology appraisal (TA) recommendations are absorbed in your Health Board?
- Do you have a “NICE reserve” or equivalent? If so, how is the level of funding determined, and where do those funds come from?

As some of the NICE technology appraisal (TA) recommendations and the scales of their financial impacts are likely to have been unexpected and so could not have been planned for at the beginning of the financial year, we would expect them to have put strain on your Health Board’s budget in-year. Think about the period since 1/10/10:

- How in practice did your Health Board find the funds to comply with NICE TAs issued since then?
- Could you identify any particular NICE TAs issued during this period which had a large budget impact?
- Were any particular services displaced, in the sense that they were discontinued, received less funding, or the referral thresholds were significantly raised in response to the financial burden imposed by a NICE TA?

*If yes, for every displaced service:*

- Where a service was displaced in order to fund a new technology recommended by NICE, did that happen within that clinical area, or are budgets reallocated across clinical areas?
- Why did you choose to pull resources from this service?
- Could you explain the processes behind the disinvestment decision? i.e. board meetings, evidence collection
- Did you use a formal prioritisation framework to help make the decision? *[Mention “All Wales Prioritisation Framework, Nov 2011]*
- What were the criteria that you assessed?
- How important was cost-effectiveness in making the displacement decision?
- How important was the cost per QALY in making the displacement decision?

How else did you respond in-year to the financial demands of new NICE TAs:

- Did you seek the additional funds needed by means of some or all of the following (and roughly in what proportions)
  - Delaying planned increases in services
  - Squeezing service providers to deliver efficiencies, i.e. the same health gain and quality of service at lower cost
  - Allocating some of the contingency funds you had available
  - Or was no specific action taken?

*If answer was that in-year “we allocated contingency funds” or “we took no specific action”, then did the NICE TAs cumulatively affect your expenditure plans for the next financial year? In what ways?*

- Did you seek the additional funds needed by means of some or all of the following (and roughly in what proportions)
  - Cutting services (i.e. disinvesting from or reducing services)
  - Delaying planned increases in services
  - Squeezing service providers to deliver efficiencies, i.e. the same health gain and quality of service at lower cost
- Where budgets had to be reallocated away from other services in order to fund a new technology recommended by NICE, did that happen within that clinical area, or are budgets reallocated across clinical areas?

Did you treat the funding demands of NICE recommendations differently from other sources of in-year financial shocks?

- Were there any other in-year financial “shocks” which required extra funds to be made available?
- How did you accommodate these shocks?

*If response is unclear:*

- Did you seek the additional funds needed by means of some or all of the following (and roughly in what proportions)
  - Cutting services (i.e. disinvesting from or reducing services)
  - Delaying planned increases in services
  - Squeezing service providers to deliver efficiencies, i.e. the same health gain and quality of service at lower cost
  - Using contingency funds you had available

*For any displaced services identified:*

- Where a service was displaced in order to fund a new technology recommended by NICE, did that happen within that clinical area, or are budgets reallocated across clinical areas?
- Why did you choose to pull resources from this service?
- Could you explain the processes behind the disinvestment? i.e. board meetings, evidence collection
- Did you use a formal prioritisation framework to help make the decision? *[Mention “All Wales Prioritisation Framework, Nov 2011]*
- What were the criteria that you assessed?
- How important was cost-effectiveness in making the disinvestment decision?
- How important was the cost per QALY in making the disinvestment decision?

**Opportunity to ask questions**

Do you have any questions about the project or the interview?

We will write a concise note of the main points from the interview, which we will send to you for confirmation or correction.

We will provide all interviewees with an early copy of the report/article we will write about this research.

Thank you for your time.
