# Supplementary material for: Opportunity costs and local health service spending decisions: a qualitative study from Wales
Source: BMC Health Serv Res. 2016 Mar 25;16:103. doi: 10.1186/s12913-016-1354-1 (PMC4807555; doi:10.1186/s12913-016-1354-1)
Supplement: Additional file 2: — COREQ checklist. (DOCX 20 kb) [file 12913_2016_1354_MOESM2_ESM.docx]

**Additional File 2: Consolidated criteria for reporting qualitative studies (COREQ), 32-item checklist^[[1]](#footnote-1)^**

| **No. Item** | **Guide questions/description** | **Addressed in manuscript/response** |
| --- | --- | --- |
| **Domain 1: Research team and reflexivity** | | |
| *Personal Characteristics* |  |  |
| 1. Interviewer/facilitator | Which author/s conducted the interview or focus group? | Reported in the “Analysis” subsection of the “Methods” section |
| 2. Credentials | What were the researcher’s credentials? E.g. PhD, MD | N/A |
| 3. Occupation | What was their occupation at the time of the study? | SKS was an Economist at OHE and JS was Deputy Director at OHE |
| 4. Gender | Was the researcher male or female? | Reported on Title Page |
| 5. Experience and training | What experience or training did the researcher have? | Experience in health services research, health policy/economics and qualitative methods |
| *Relationship with participants* |  |  |
| 6. Relationship established | Was a relationship established prior to study commencement? | Reported in the “Interview targets” subsection of the “Methods” section |
| 7. Participant knowledge of the interviewer | What did the participants know about the researcher? e.g. personal goals, reasons for doing the research | Reported in “Additional File 1” |
| 8. Interviewer characteristics | What characteristics were reported about the interviewer/facilitator? e.g. Bias, assumptions, reasons and interests in the research topic | Reported in “Additional File 1” |
|  | | |
| *Theoretical framework* |  |  |
| 9. Methodological orientation and Theory | What methodological orientation was stated to underpin the study? e.g. grounded theory, discourse analysis, ethnography, phenomenology, content analysis | Reported in the “Analysis” subsection of the “Methods” section |
| *Participant selection* |  |  |
| 10. Sampling | How were participants selected? e.g. purposive, convenience, consecutive, snowball | Reported in the “Interview targets” subsection of the “Methods” section |
| 11. Method of approach | How were participants approached? e.g. face-to-face, telephone, mail, email | Reported in the “Interview targets” subsection of the “Methods” section |
| 12. Sample size | How many participants were in the study? | Reported in the “Interview targets” subsection of the “Methods” section |
| 13. Non-participation | How many people refused to participate or dropped out? Reasons? | Reported in the “Interview targets” subsection of the “Methods” section |
| *Setting* |  |  |
| 14. Setting of data collection | Where was the data collected? e.g. home, clinic, workplace | Workplace |
| 15. Presence of nonparticipants | Was anyone else present besides the participants and researchers? | No |
| 16. Description of sample | What are the important characteristics of the sample? e.g. demographic data, date | Reported in the “Interview targets” subsection of the “Methods” section |
| *Data collection* |  |  |
| 17. Interview guide | Were questions, prompts, guides provided by the authors? Was it pilot tested? | Interview guide reported in “Additional File 1”. No pilot |
| 18. Repeat interviews | Were repeat interviews carried out? If yes, how many? | No |
| 19. Audio/visual recording | Did the research use audio or visual recording to collect the data? | Yes, reported in the “Interview structure” subsection of the “Methods” section |
| 20. Field notes | Were field notes made during and/or after the interview or focus group? | Yes, reported in the “Interview targets” subsection of the “Methods” section |
| 21. Duration | What was the duration of the interviews or focus group? | Reported in the “Interview targets” subsection of the “Methods” section |
| 22. Data saturation | Was data saturation discussed? | No |
| 23. Transcripts returned | Were transcripts returned to participants for comment and/or correction? | Yes, reported in the “Interview structure” subsection of the “Methods” section |
|  | | |
| *Data analysis* |  |  |
| 24. Number of data coders | How many data coders coded the data? | Reported in the “Analysis” subsection of the “Methods” section |
| 25. Description of the coding tree | Did authors provide a description of the coding tree? | No |
| 26. Derivation of themes | Were themes identified in advance or derived from the data? | Reported in the “Analysis” subsection of the “Methods” section |
| 27. Software | What software, if applicable, was used to manage the data? | Microsoft Word/Excel |
| 28. Participant checking | Did participants provide feedback on the findings? | No |
| *Reporting* |  |  |
| 29. Quotations presented | Were participant quotations presented to illustrate the themes/findings? Was each quotation identified? e.g. participant number | Yes, reported in “Results” section |
| 30. Data and findings consistent | Was there consistency between the data presented and the findings? | Yes |
| 31. Clarity of major themes | Were major themes clearly presented in the findings? | Yes |
| 32. Clarity of minor themes | Is there a description of diverse cases or discussion of minor themes? | Yes |

1. Tong, A., Sainsbury, P., and Craig, J. (2007). Consolidated criteria for reporting qualitative research (COREQ): a 32-item checklist for interviews and focus groups. *International Journal for Quality in Health Care*, *19*(6), 349. [↑](#footnote-ref-1)
